# Regional differences in the management of patients with mild traumatic brain injury and antithrombotic therapy—an Austrian survey

**DOI:** 10.1007/s00402-026-06339-8

**Published:** 2026-05-25

**Authors:** Barbara Sebek, Dietmar Dammerer, David Putzer, Lukas Moser

**Affiliations:** 1 Department of Orthopaedics and Traumatology, University Hospital Krems – NOE LGA, Karl Landsteiner University, Mitterweg 10, 3500 Krems, Austria; 2 University for Continuing Education Krems, Krems, Austria; 3 Innsbruck Medical University, Innsbruck, Austria

**Keywords:** Traumatic brain injury, Head trauma, Oral anticoagulants, Anticoagulation

## Abstract

**Background/purpose:**

The number of patients with mild traumatic brain injury (TBI) receiving oral anticoagulation is increasing. Up-to-date diagnostic and treatment algorithms are missing in Austria. The aim of this survey is to collect and analyse Austria-wide data on the care of this patient group.

**Methods:**

The survey was carried out using “Jotform” between 27 November 2023 and 31 January 2024, asking questions relating to the diagnosis and treatment of patients with mild TBI receiving antithrombotic therapy. Patient groups comprising individuals with alcohol addiction and hemophilia were incorporated as well with a view to the paucity of available literature on these groups. The survey was sent to all orthopedic-traumatological and neurosurgical departments in Austria.

**Results:**

39 of 67 (58.2%) orthopedics & traumatology departments and 4 of 10 (40%) neurosurgery departments participated in the survey. The share of departments reporting routine submission of different patient groups to cranial computed tomography (CCT) was 38 (95%) for patients taking antiplatelet agents, 39 (97.5%) departments for patients taking vitamin K antagonists (VKAs), 39 (97.5%) for patients taking direct oral anticoagulants (DOACs), 11 (27.5%) for patients with alcohol addiction, and 28 (70%) for patients with known hemophilia. Considering the separate groups of patients not living in a nursing facility vs. those living in a nursing facility, inpatient admission occurred for 63% vs. 44% of patients taking antiplatelet agents, for 78% vs. 61% of patients taking VKAs, 80.5% vs. 63.4% of patients taking DOACs, 17.1% vs. 17% of patients with alcohol addiction, and 51.2% vs. 43.9% of patients with hemophilia. Statistically significant regional differences in inpatient admission were found in the group with hemophilia (living and not living in a nursing facility) as well as in the group with DOAC intake (living in a nursing facility).

**Conclusions:**

Regional differences in the treatment of the studied patient groups exist within Austria, particularly regarding in-house admission of patients. An up-to-date guideline would be desirable to enable the best possible patient care while taking the increasing resource scarcity into account.

**Supplementary Information:**

The online version contains supplementary material available at 10.1007/s00402-026-06339-8.

## Introduction

The incidence of traumatic brain injury (TBI) in Europe has been estimated to reach up to 849 cases per 100,000 population per year, although considerable variability between regions has been reported [1]. In the German-speaking region, an epidemiological study published in 2010 reported an incidence of 332 cases per 100,000 inhabitants in Germany, with healthcare costs estimated at approximately 2.8 billion euros annually [2]. Data from the pilot phase of a trauma database for German-speaking countries published in 2023 showed that 40.5% of patients presented with mild TBI according to the Glasgow Coma Scale. Falls represented the most common mechanism of injury (56.8%), and the mean patient age was 54.7 ± 23.2 years. Notably, 26.3% of patients were receiving oral anticoagulation therapy at the time of their injury [3]. Intracranial hemorrhage (ICH) represents the most threatening complication of TBI and can only be reliably diagnosed via cranial computed tomography (CCT) [4]. Although treatment algorithms have been developed in other regions, such as the “Scandinavian guidelines for initial management of minimal, mild, and moderate head injuries in adults” [5], substantial disagreement concerning the optimal management of these patients remains. No comprehensive national guideline providing diagnostic and therapeutic algorithms for TBI currently exists in Austria. There is particular uncertainty regarding the management of patients receiving direct oral anticoagulants (DOACs), which have largely replaced vitamin K antagonists (VKAs) in clinical practice. Available evidence suggests that immediate ICH occurs more frequently in patients treated with VKAs than in those receiving DOACs, whereas the incidence of delayed ICH appears to be similar between the two groups [6]. An AWMF (Arbeitsgemeinschaft der Wissenschaftlichen Medizinischen Fachgesellschaften) guideline on TBI in adulthood published in 2015 is currently undergoing revision [7]. In Austria, a national consensus statement published in 2019 provides practical recommendations for the management of this patient population; however, it also highlights the lack of robust evidence required to formulate definitive treatment recommendations [8]. Given the increasing strain on healthcare systems [9], efficient and evidence-based management of patients with mild TBI is of growing importance. The literature presents heterogeneous findings regarding the risk and outcomes of mild TBI in patients receiving oral anticoagulation. For example, Antoni et al. reported a probability of 1.2% for developing ICH during inpatient observation following mild TBI [10]. Fiorelli et al. reported a higher risk of ICH following mild TBI in patients receiving antiplatelet therapy compared with patients not receiving antithrombotic medication, although no differences were observed with regard to mortality or the need for neurosurgical intervention [11]. A prospective study published in 2020 that investigated patients with mild TBI receiving DOAC therapy reported an overall risk of adverse outcomes of 3.4%. Based on these low event rates, the authors suggest that shared decision-making between physician and patient may be preferable to routine imaging after minor head injury in this population [12]. Similarly, Santing et al. demonstrated a low risk of ICH in patients with TBI receiving DOAC therapy [6]. A systematic review and meta-analysis published in 2022 found that DOAC use was associated with a lower risk of traumatic ICH compared with VKAs, while the risk was comparable to that observed in patients receiving antiplatelet therapy. Patients treated with DOACs required reversal agents and neurosurgical interventions less frequently than those receiving VKAs. By contrast, neurosurgical intervention rates did not differ significantly between patients receiving DOACs, antiplatelet therapy, or no antithrombotic treatment. Rates of ICH progression, delayed ICH, and TBI-related in-hospital mortality were similar across all treatment groups. Overall, these findings suggest that following mild TBI, elderly patients receiving DOAC therapy have a lower risk of adverse outcomes than those treated with VKAs, and a risk comparable to that of patients receiving antiplatelet therapy [13]. Similarly, Liu et al. reported no statistically significant differences between patients receiving VKAs and those receiving DOACs with respect to mortality, need for neurosurgical intervention, progression of ICH, or length of hospital and intensive care stay. These results suggest that clinical outcomes in elderly TBI patients are comparable between the two treatment groups [14]. Despite ongoing debate regarding the risk of ICH, computed cranial tomography remains the diagnostic modality used in nearly all clinical studies. Some authors have questioned the benefit of routine imaging, however Colas et al. assessed the value of systematic CCT in patients with mild TBI receiving antithrombotic therapy, including antiplatelet agents, VKAs, and DOACs. Although some cases of ICH were detected, the overall rate of clinically significant findings and interventions was low. The authors therefore suggest that routine imaging of all such patients may offer limited benefit, and that a more selective diagnostic approach could be sufficient [15]. Overall, the current literature consistently highlights the need for further prospective studies to allow more definitive conclusions regarding the optimal management of mild TBI in patients receiving antithrombotic therapy [11–15]. 

Beyond individual risk factors, differences in healthcare systems may also affect management strategies and outcomes. A nationwide Swedish cohort study using data from the Swedish Trauma Register identified substantial regional variation in TBI patterns, treatment strategies, and mortality rates [16]. Similarly, a European survey demonstrated considerable variability among neurotrauma centres in the implementation and adherence to guidelines for severe TBI management [17]. 

In discussions with colleagues across Austria, substantial variability in the management of patients with mild TBI receiving antithrombotic therapy has also been observed, particularly regarding the decision between inpatient admission and discharge after CCT imaging. The aim of the present study was therefore to quantify this variability in clinical practice.

## Materials and methods

The online survey was carried out between 27 November 2023 and 31 January 2024 using “Jotform” (https://www.jotform.com/). The questions shown in the following table were sent to all 67 orthopedic-traumatological and 10 neurosurgical departments in Austria.

**Table 1:** Questionnaire consisting of 9 questions

Please indicate the specialization of your department – orthopedics & traumatology or neurosurgery. In which federal state do you work? Please specify your institution.
Is there an in-house standard for the treatment of patients with traumatic brain injury and intake of oral anticoagulation/antiplatelet medication? If yes: Is the in-house standard available in written form to all department members? *In-house standard*
Which patients receive a cranial computed tomography (CCT) for diagnosis? If none of the mentioned patient groups: Which patients are instead subjected to inpatient observation (regular vigilance, pupil, and blood pressure checks) WITHOUT undergoing a CCT (living/not living in a nursing facility)? *CCT for diagnosis*
When is the CCT performed (assuming it would be available immediately)? *Timing of CCT*
Which patients (NOT living in a nursing facility) are subject to inpatient observation (regular vigilance, pupil, and blood pressure checks) after a normal CCT? *Inpatient observation for patients living at home*
Which patients (living in a nursing facility) are subject to inpatient observation (regular vigilance, pupil, and blood pressure checks) after a normal CCT? *Inpatient observation for patients living in a nursing facility*
Do patients with normal CCT scans who are admitted for inpatient observation routinely undergo a follow-up CCT scan after 24 h? *Routinely follow-up CCT after 24 h after normal CCT*
Is S100B blood testing routinely performed in cases of isolated mild traumatic brain injury? *S100B for mild traumatic brain injury*
Do patients with a history of alcohol consumption or known alcohol addiction routinely have their coagulation parameters determined? *Alcohol consumption and coagulation*
Are you familiar with the “Scandinavian guidelines for initial management of minimal, mild, and moderate head injuries in adults”? If yes: Do you work according to these guidelines? *Familiarity with the Scandinavian Guidelines*

The survey link was sent by email to the department heads of all orthopedics & traumatology and neurosurgery departments in Austria on 27 November 2023 with a request to return one answer per department. Two reminders were added with a one-time extension of the deadline to increase the response rate. The survey was finally completed on 31 January 2024. The contact details and list of departments were taken from the website of the Austrian Society for Trauma Surgery (https://www.unfallchirurgen.at/patienteninformationen/krankenhaeuser-und-ordinationen/) and the Austrian Society for Neurosurgery (https://www.neurochirurgie.ac.at/kliniken-abteilungen).

The questionnaires were evaluated using descriptive statistics and crosstabs. The frequencies and percentage distributions were calculated. Fisher’s exact test (one-sided) was used to determine differences between each group. A *p*-value of 0.05 was assumed to be statistically significant. All analyses were performed using SPSS (version 29.0, IBM Corp., Armonk, NY).

## Results

Of the total of 67 orthopedic-traumatological and 10 neurosurgical departments, 39 (58.2%) orthopedic-traumatological and 4 (40%) neurosurgical departments responded to the survey, resulting in a general response rate of 43 replies (55.8%). More than one reply was received from two orthopedics & traumatology departments; in one case there were two answers, in another there were five. The answers from the multiple respondents were compared with each other. Identical answers were combined into one; different answers from a single department were considered invalid and consequently excluded from the statistical analysis. Furthermore, some questions were omitted by a department or answered with text and therefore could not be assigned to any possible answer, which is why the numerical number of answers differs for some questions. Both the numerical and percentage figures are always provided in this regard. Due to the predominantly orthopedic-traumatological feedback, the following results should be interpreted with caution, as a higher response rate from neurosurgical departments might have led to different results.

**Fig. 1:**
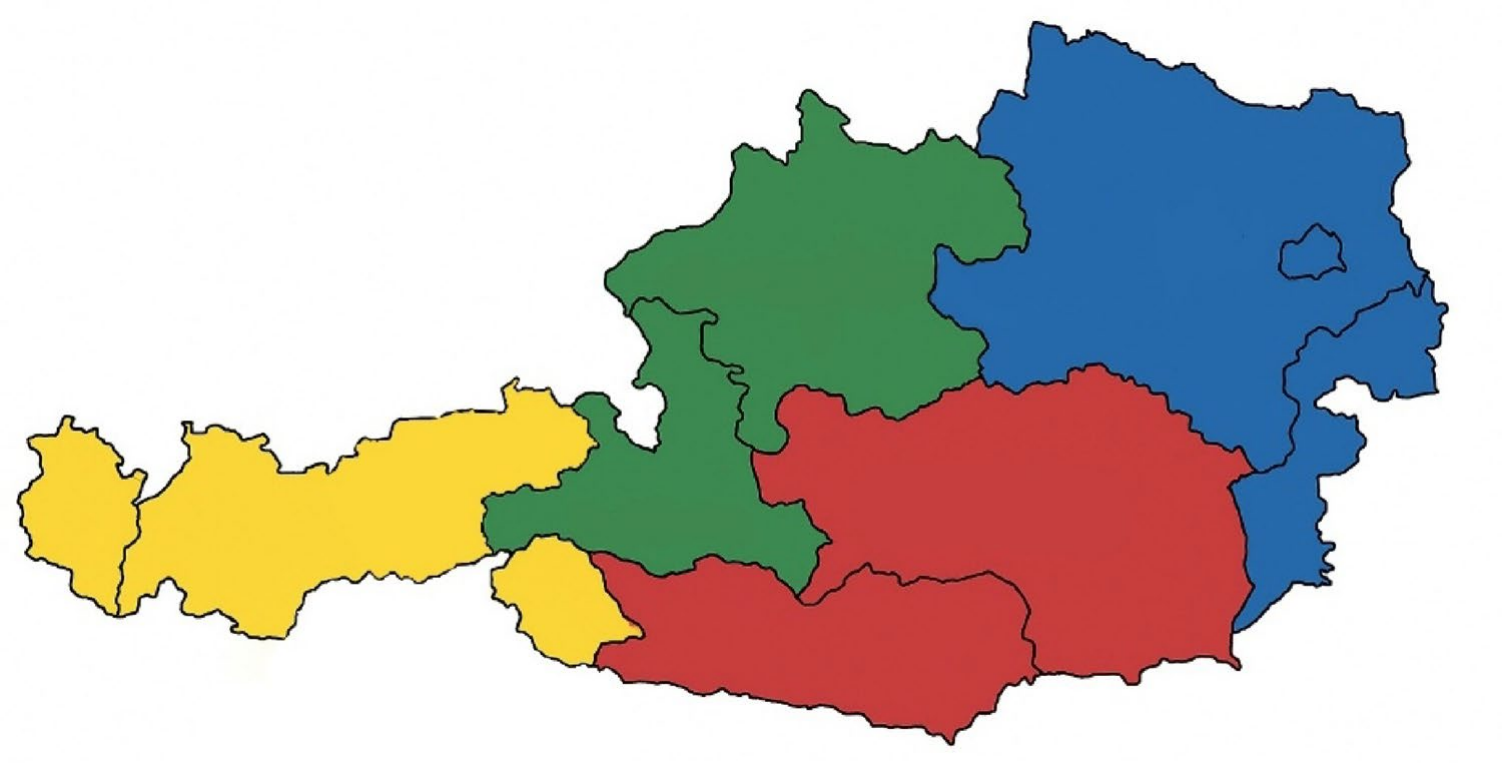
Region “West” (Vorarlberg, Tyrol; yellow), region “Central” (Salzburg, Upper Austria; green), region “South” (Styria, Carinthia; red), region “East” (Lower Austria, Vienna, Burgenland; blue)

In order to present the responses by region and compare them with each other, they were divided into four regions: “West” with the federal states of Vorarlberg and Tyrol, “Central” with Salzburg and Upper Austria, “South” with Styria and Carinthia, and “East” with Lower Austria, Vienna, and Burgenland. There were 7 responses (16.3%) from the Western region, 11 responses (25.6%) from the Central region, 8 responses (18.6%) from the Southern region, and 17 responses (39.5%) from the Eastern region (Fig. 1).

### In-house standard

In 34 (79.1%) departments, there was an in-house standard, while 5 (11.6%) reported no standard. The departments with an in-house standard were asked whether the standard was also available in written form. A written version was available in 25 departments (73.5%), while 9 (26.5%) reported no documentation. No statistically significant differences were found between the regions regarding the existence of in-house standards (*p* = 0.707).

Table 2 contains cross-tabulations of inpatient admission variables based on type of oral anticoagulation taken by patients compared to the presence of a standard practice in the same department. Although low *p*-values ​​are evident for patients not living in a nursing facility and taking VKAs and DOACs, no significant difference in the frequency of inpatient admissions was observed depending on whether or not a standard practice was in place in the department.

**Table 2:** Inpatient admission variables by oral anticoagulation type versus departmental standard practice - Crosstabs reporting *p*-values

	Standard	Inpatient admission (patients not living in nursing facility)			Inpatient admission (patients living in nursing facility)		
		Yes	No	*p*-value	Yes	No	*p*-value
Antiplatelet agent	Yes	21 (62%)	13 (38%)	0.326	16 (47%)	18 (53%)	0.262
	No	2 (40%)	3 (60%)		1 (20%)	4 (80%)	
Vitamin K antagonist	Yes	28 (82%)	6 (18%)	0.070	23 (67%)	11 (33%)	0.237
	No	2 (40%)	3 (60%)		2 (40%)	3 (60%)	
DOAC	Yes	27 (79%)	7 (21%)	0.096	22 (65%)	12 (35%)	0.280
	No	2 (40%)	3 (60%)		2 (40%)	3 (60%)	

### CCT for diagnosis

A CCT was performed after head injury on 95% (38) of patients taking antiplatelet agents, 97.5% (39) of patients taking VKAs, 97.5% (39) of patients taking DOACs, 27.5% (11) of patients with known regular alcohol consumption or known alcohol addiction, and 70% (28) of patients with known hemophilia (*n* = 40).

### Timing of CCT

In 97.6% (41), the CCT was carried out immediately. Only one institution (2.4%) routinely performed the CCT the following morning (*n* = 42).

### Inpatient observation

**Fig. 2:**
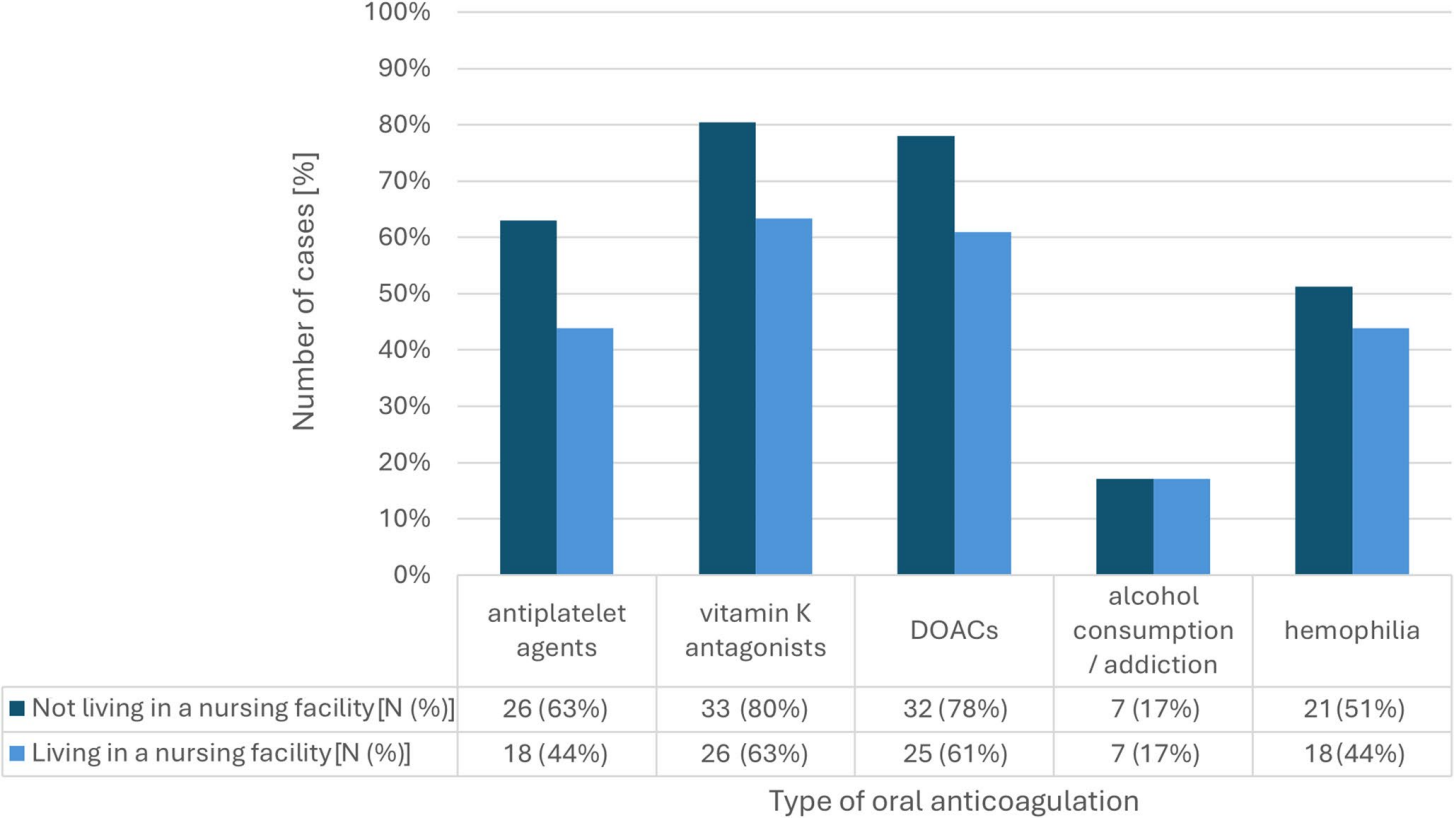
Patients admitted to inpatient observation (regular vigilance, pupil, and blood pressure checks) after a normal CCT (n = 41)

Figure 2 shows the percentage of departments reporting admission of patients as inpatients. Patients living at home were admitted as inpatients more frequently than those living in a nursing facility. Furthermore, patients with regular alcohol consumption or known alcohol addiction were generally admitted as inpatients less frequently than the other studied groups.

### Inpatient observation for patients living at home

Regional differences concerning inpatient observation of patients not living in a nursing facility are presented in Table 3.

No significant regional differences were found regarding patients taking antiplatelet agents, VKAs, and DOACs, as well as regarding patients with regular alcohol consumption/addiction. Patients with hemophilia were significantly more likely to be admitted for inpatient observation in the Central region compared to the Southern region (72.7% vs. 14.3%, *p* = 0.025). P-values are shown in Table 4.

**Table 3:** Patients (NOT living in a nursing facility) admitted for inpatient observation (regular vigilance, pupil, and blood pressure checks) after a normal CCT - Responses from the different regions (*n* = 42).

	Antiplatelet agent	Vitamin K antagonist	DOAC	Alcohol consumption / addiction	Hemophilia
West	4 (57%)	4 (57%)	4 (57%)	2 (29%)	3 (43%)
Central	9 (82%)	10 (91%)	10 (91%)	4 (36%)	**8 (73%)**
South	3 (43%)	5 (71%)	4 (57%)	0 (0%)	**1 (14%)**
East	10 (59%)	14 (82%)	14 (82%)	1 (6%)	9 (53%)

**Table 4:** Patients (NOT living in a nursing facility) admitted for inpatient observation (regular vigilance, pupil, and blood pressure checks) after a normal CCT - Crosstabs reporting *p*-values.

	West	Central	South	East
Antiplatelet agent				
West	-	0.272	0.500	0.643
Central		-	0.117	0.197
South			-	0.395
East				-
Vitamin K antagonist				
West	-	0.137	0.500	0.215
Central		-	0.326	0.482
South			-	0.462
East				-
DOAC				
West	-	0.137	0.704	0.215
Central		-	0.137	0.482
South			-	0.215
East				-
Alcohol consumption / addiction				
West	-	0.572	0.231	0.194
Central		-	0.108	0.062
South			-	0.708
East				-
Hemophilia				
West	-	0.220	0.280	0.500
Central		-	**0.025**	0.260
South			-	0.097
East				-

### Inpatient observation for patients living in a nursing facility

Regional differences concerning inpatient observation for patients living in a nursing facility are presented in Table 5.

Patients receiving DOACs were significantly more likely to be admitted for inpatient observation in the Central region compared to the Southern region (82% vs. 29%; *p* = 0.039). Patients with hemophilia were likewise significantly more likely to be admitted for inpatient observation in the Central region compared to the Southern region (64% vs. 47%; *p* = 0.01) (Tables 5 and 6).

**Table 5:** Patients (living in a nursing facility) admitted for inpatient observation (regular vigilance, pupil, and blood pressure checks) after a normal CCT - Responses from the different regions (*n* = 42).

	Antiplatelet agent	Vitamin K antagonist	DOAC	Alcohol consumption / addiction	Hemophilia
West	3 (43%)	3 (43%)	3 (43%)	2 (29%)	3 (43%)
Central	7 (64%)	9 (82%)	**9 (82%)**	4 (36%)	**7 (64%)**
South	1 (14%)	3 (43%)	**2 (29%)**	0 (0%)	0 (0%)
East	7 (41%)	11 (65%)	11 (65%)	1 (6%)	**8 (47%)**

**Table 6:** Patients (living in a nursing facility) admitted for inpatient observation (regular vigilance, pupil, and blood pressure checks) after a normal CCT - Crosstabs reporting *p*-values.

	West	Central	South	East
Antiplatelet agent				
West	-	0.352	0.280	0.643
Central		-	0.057	0.220
South			-	0.218
East				-
Vitamin K antagonist				
West	-	0.117	0.704	0.296
Central		-	0.117	0.296
South			-	0.296
East				-
DOAC				
West	-	0.117	0.500	0.296
Central			**0.039**	0.296
South				0.122
East				-
Alcohol consumption / addiction				
West	-	0.572	0.231	0.194
Central		-	0.108	0.062
South			-	0.708
East				-
Hemophilia				
West	-	0.352	0.096	0.605
Central		-	**0.010**	0.320
South			-	0.033
East				-

### Routine follow-up CCT after 24 h following normal CCT

In 6 departments (14.6%), a follow-up CCT was routinely performed after an initial normal CCT. In 35 departments (85.4%), no follow-up CCT was routinely performed. No significant regional differences were determined (*p* = 0.136) (*n* = 41).

### S100B for mild traumatic brain injury

Among patients with isolated mild TBI, S100B was routinely assessed in 7 departments (17.1%) and not routinely assessed in 34 departments (82.9%). No significant regional differences were determined (*p* = 0.610) (*n* = 41).

### Alcohol consumption and coagulation

Coagulation parameters were routinely measured in 16 departments (38.1%) in patients with regular alcohol consumption or known alcohol addiction, whereas 24 departments (57.1%) did not routinely measure coagulation parameters. In 2 departments (4.8%), the coagulation parameters were only measured if the patient was admitted. No significant regional differences were determined (*p* = 0.610) (*n* = 42).

### Familiarity with the Scandinavian Guidelines

20 departments (48.8%) were aware of the “Scandinavian guidelines for initial management of minimal, mild, and moderate head injuries in adults”. Of these, 5 departments (25%) followed the guidelines and 13 departments (65%) did not. 21 departments (51.2%) were unaware of the Scandinavian Guidelines. No significant regional differences were determined regarding the application of the guidelines (*p* = 0.055) (*n* = 41).

## Discussion

The most important finding of the present study are differences regarding inpatient admission of patients with TBI and oral anticoagulation. Differences were determined both between individual oral anticoagulants and between patients living in a nursing facility and patients living at home. Differences were also observed when comparing individual regions, although no statistically significant differences were observed in the group of patients not living in a nursing facility.

As expected, the study showed fewer inpatient admissions among patients cared for by nursing staff. When patients lived in a nursing home, inpatient care was more often foregone and observation handed over to the nursing staff. This circumstance would warrant a discussion of the increasing resource scarcity in nursing homes, which likely do not allow the same level of observation as in a hospital, but no scientific data on this is available. Another ethically questionable argument for this approach is probably the older age and poorer general condition of these patients, since even in the case of post-traumatic ICH, there are hardly any indications for interventions.

Furthermore, it should be kept in mind that inpatient admission usually ends after 24 h, whereas ICH sometimes occurs days or weeks after trauma [10]. 

A comparison of the various oral anticoagulants showed the lowest rate of inpatient admission for those taking platelet inhibitors compared to VKAs and DOACs (63% vs. 78% vs. 80% for patients living at home; 44% vs. 61% vs. 63% for patients living in a nursing facility). This can be justified in part with reference to the consensus paper published in 2019 by Wiegele et al., in which discharge is indicated as a possible option for patients under mono antiplatelet therapy and GCS 15 who are under guaranteed care by nursing staff or instructed relatives [8]. The study shows hardly any differences between VKAs and DOACs, although DOACs are even less researched in the literature than VKAs.

The most relevant question of this study was to identify geographical differences in treatment, particularly in inpatient admissions. Although no statistically significant differences were found, differences regarding patients not living in a nursing facility were determined – particularly between the Central region, where most departments routinely admitted patients (antiplatelet agents 82%, VKA 91%, DOAC 91%), and the Southern region, where inpatient admissions were much less common (antiplatelet agents 43%, VKA 71%, DOAC 57%). Patients living in a nursing facility were admitted to hospital significantly more often in the Central region than in the Southern region if they were also taking DOACs (82% vs. 29%). In summary, the Central region was the most cautious and admitted patients most frequently, though the reasons why remain unknown. Further studies or surveys would be needed to determine the factors affecting inpatient admission thresholds. Possible reasons for the differing results may include the lack of current guidelines or in-house standards, though this is only speculation. Other aspects such as differing hospital structures, especially including access to neurosurgery (available in only 10 hospitals in Austria) and intensive care beds, may also influence patient admissions. Cultural differences in medical practice regarding admission thresholds may also contribute to the diverging results.

Patients with regular alcohol consumption/known alcohol addiction or known hemophilia and mild TBI are treated differently in Austria. The study shows the highest admission rates (for patients living at home vs. patients living in a nursing facility) in the Central region (alcohol 36.4% vs. 36.4%; hemophilia 72.7% vs. 63.3%). In the Southern region, inpatient admission did not occur for patients with alcohol addiction (both living and not living in a nursing facility) or for patients with hemophilia living in a nursing facility. Only patients with hemophilia living at home were subject to inpatient admission in 14.3% of departments in this region. The data showed that patients with hemophilia, which at approximately 800 affected individuals represent only a very small percentage of the population in Austria [18], as well as patients with alcohol addiction, which represent 5% of the population [19], are not subject to uniform diagnostic and treatment algorithms. This is likely due to the scarcity of available literature on the one hand and to the small number of patients with hemophilia in everyday clinical practice on the other.

Patients under the influence of alcohol often exhibit limited neurological examinability and compliance, and evaluating their medical history can be difficult as well. This can significantly complicate the assessment of the severity of a TBI. It can also lead to different treatment of this patient group, as well as to the treating physician misjudging the situation and ICH being overlooked as a result. In both hemophilia patients and patients with alcohol addiction who are not obviously intoxicated at the time of injury, it is also important to note that a medical history evaluation taking these two possibilities into consideration is certainly not always carried out as standard practice.

Another important finding of this survey concerns the topic of CCTs and follow-up CCTs. An initial CCT was performed on patients taking antiplatelet agents in 95% of departments, on patients taking VKAs in 97.5% of departments, and on patients taking DOACs in 97.5% of departments. Cranial computed tomography remains the gold standard for detecting ICH in patients with oral anticoagulation intake, and this is reflected in the survey results. While Colas et al. question the necessity of CCT, they deliver no clear recommendation against it either, calling for more studies on the topic [15]. Despite this, only 27.5% of patients with known regular alcohol consumption or known alcohol addiction and 70% of patients with known hemophilia received a CCT. Due to the impaired coagulation in these groups, a stricter indication for performing a CCT would likely be appropriate. Regarding the timing of the CCT, almost all surveyed departments reported performing it immediately. However, there is broader consensus in the current literature and the 2019 consensus paper on the topic of the standard follow-up CCT, which is not recommended if no neurological abnormalities are found during observation [8, 15, 10, 20, 21]. In the survey, 14.6% of responding departments performed a standard follow-up CCT after a normal initial CCT.

An in-house standard was available in 79.1% of all departments, which is positive, as standards prevent errors. However, since this standard was not available in writing in 26.5% of cases, errors may still occur in these departments. Blood coagulation testing was routinely performed at only 38.1% of the departments. While there are no recommendations for coagulation tests in patients with alcohol addiction and mild TBI to be found in the literature, it would be interesting to know whether pathological or physiological coagulation also has an impact on CCT or inpatient admission decisions in the departments with standard coagulation testing. Further studies and surveys will be necessary. Furthermore, we sought to determine whether the presence of an in-house standard promotes greater consistency in clinical practice. To this end, we compared the implementation of inpatient admission procedures for patients receiving different oral anticoagulants, distinguishing between those living at home and those residing in nursing facilities. The presence of a standard practice in a given department did not correlate with significant differences in the frequency of inpatient admissions (both living at home and living in a nursing facility) based on the type of oral anticoagulation, and a more consistent practice can therefore not be assumed under these circumstances. However, a limitation here is that there are significantly more departments with a standard practice than without one, which restricts the generalizability of this result.

In this study, 48.8% of all responding departments reported being aware of the “Scandinavian guidelines for initial management of minimal, mild, and moderate head injuries in adults”, but only 25% followed them. The survey explicitly asked about this particular guideline, which appears to lack acceptance in Austria in light of these figures. S100B testing was only performed by 17.1% of departments. The Scandinavian Guidelines include the determination of S100B in the blood of patients with isolated mild TBI with a trauma during the last six hours to gauge the need for a CCT [22]. The study by Rahimian et al. also mentions the potential for S100B determination to reduce the number of unnecessary CCTs in intoxicated patients [23]. However, limitations to performing this blood test include the essential absence of additional injuries and the longer duration of the procedure compared to a CCT scan. Moreover, in the absence of a CCT, ICH cannot be definitively excluded. Consequently, despite its recommendation in the Scandinavian Guidelines, S100B testing is likely to remain unpopular.

The present study has a considerable number of limitations. To achieve the highest possible response rate and conserve the time resources of the department heads asked to participate, granular response options and more precise details were omitted. First, the survey did not ask to specify the duration of observation nor whether – as in my department – it ended after at least 24 h. Second, the time interval between vigilance, pupil, and blood pressure checks was not queried. Third, information about which department (orthopedics & traumatology, neurosurgery, internal medicine, geriatrics) the patients were admitted to was not collected. Fourth, we did not ask about other guidelines besides the Scandinavian Guidelines (nor was this question formulated as an open question) for the reasons stated above. Overall, the response rate was low at 55.8% across all orthopedic-traumatological and neurosurgical departments in Austria; the data from this survey should therefore be interpreted with caution. The possibility of a non-response bias should be mentioned in this context. With most of the responses coming from orthopedic-traumatological departments, a clear underrepresentation of the neurosurgical perspective is evident. A higher response rate from neurosurgical departments might have yielded different results. Therefore, the results cannot be generalized, nor is an accurate representation of all non-responding departments possible – whether orthopedics & traumatology or neurosurgery. In addition, the limitation arising from the reliance on self-reported practices should be acknowledged, as the responses may not accurately reflect actual behaviours given that they are based on the perceptions of only one individual per department. Nevertheless, this survey is the first in Austria to examine the different diagnostic and treatment strategies for the specified patient group and provides valuable insight into the topic.

## Conclusion

In summary, the results of this survey demonstrate the general and geographical differences in diagnostics and treatment (and by extension, the need for uniform guidelines for diagnostics and treatment) of patients with mild TBI who are under oral anticoagulation or known to suffer from hemophilia or alcohol addiction, with the aim of enabling the best possible care while also conserving resources for the healthcare system. Only clear, up-to-date guidelines offer the opportunity for uniform and targeted treatment, supporting decision-making for young doctors who primarily work in the orthopedics & traumatology or neurosurgery emergency departments in Austria.

## Supplementary Information

Below is the link to the electronic supplementary material.

Survey (german)

## Data Availability

No datasets were generated or analysed during the current study.
