# Supplementary material for: Regional differences in the management of patients with mild traumatic brain injury and antithrombotic therapy—an Austrian survey
Source: Arch Orthop Trauma Surg. 2026 May 25;146(1):200. doi: 10.1007/s00402-026-06339-8 (PMC13201303; doi:10.1007/s00402-026-06339-8)
Supplement: Supplementary file 1 — Survey (german) [file 402_2026_6339_MOESM1_ESM.pdf]

In order to submit this form, you should open it with Adobe Acrobat Reader.

Regionale Unterschiede im Management des leichten Schädel-Hirn-Traumas unter antithrombotischer Therapie

Eine österreichweite Umfrage

Bitte geben Sie die Fachrichtung Ihrer Abteilung an

Bitte auswählen

Bitte geben Sie Ihre neurochirurgische Institution an

Bitte auswählen

In welchem Bundesland sind Sie tätig?

- ○ Burgenland
- ○ Kärnten
- ○ Niederösterreich
- ○ Oberösterreich
- ○ Salzburg
- ○ Steiermark
- ○ Tirol
- ○ Vorarlberg
- ○ Wien

Burgenland - Bitte geben Sie Ihre Institution an

- ○ Krankenhaus der Barmherzigen Brüder Eisenstadt
- ○ Klinik Oberwart

Kärnten - Bitte geben Sie Ihre Institution an

- ○ Unfallkrankenhaus Klagenfurt
- ○ Klinikum Klagenfurt/Wörthersee
- ○ A.ö. Krankenhaus des Deutschen Ordens Friesach
- ○ Landeskrankenhaus Wolfsberg
- ○ Landeskrankenhaus Villach
- ○ Krankenhaus Spittal/Drau

Niederösterreich - Bitte geben Sie Ihre Institution an

- ○ Landesklinikum Amstetten
- ○ Landesklinikum Baden-Mödling
- ○ Landesklinikum Hainburg
- ○ Landesklinikum Korneuburg
- ○ Landesklinikum Mistelbach-Gänserndorf
- ○ Landesklinikum Mostviertel Waidhofen/Ybbs
- ○ Landesklinikum Neunkirchen
- ○ Landesklinikum Wiener Neustadt
- ○ Landesklinikum Waldviertel Horn
- ○ Universitätsklinikum Krems
- ○ Universitätsklinikum St. Pölten
- ○ Universitätsklinikum Tulln

Oberösterreich - Bitte geben Sie Ihre Institution an

- ○ Kepler Universitätsklinikum Linz
- ○ Unfallkrankenhaus Linz
- ○ Pyhrn-Eisenwurzen Klinikum Steyr
- ○ Pyhrn-Eisenwurzen Klinikum Kirchdorf
- ○ Klinikum Wels-Grieskirchen
- ○ Klinikum Schärding
- ○ Salzkammergut Klinikum Gmunden
- ○ Salzkammergut Klinikum Bad Ischl
- ○ Salzkammergut Klinikum Vöcklabruck
- ○ Krankenhaus Sankt Josef Braunau

Create your own automated PDFs with Jotform PDF Editor- It's free

Jotform

- ◯ Klinikum Freistadt
- ◯ Klinikum Rohrbach
- ◯ Krankenhaus Barmherzige Schwestern Ried

Salzburg - Bitte geben Sie Ihre Institution an

- ◯ Landeskrankenhaus Salzburg
- ◯ Landesklinik Tamsweg
- ◯ Tauernklinikum Zell am See
- ◯ Unfallkrankenhaus Salzburg
- ◯ Kardinal Schwarzenberg Klinikum

Steiermark - Bitte geben Sie Ihre Institution an

- ◯ LKH-Univ. Klinikum Graz
- ◯ UKH Steiermark, Standort Kalwang
- ◯ LKH Murtal, Standort Judenburg
- ◯ Klinik Diakonissen Schladming
- ◯ UKH Steiermark, Standort Graz
- ◯ LKH Hochsteiermark, Standort Bruck an der Mur
- ◯ LKH Feldbach - Fürstenfeld

Tirol - Bitte geben Sie Ihre Institution an

- ◯ A.ö. Landeskrankenhaus - Universitätskliniken Innsbruck
- ◯ Bezirkskrankenhaus Schwaz
- ◯ Bezirkskrankenhaus St. Johann in Tirol
- ◯ Bezirkskrankenhaus Reutte
- ◯ Landeskrankenhaus Hall
- ◯ Bezirkskrankenhaus Kufstein
- ◯ Krankenhaus St. Vinzenz Zams
- ◯ Bezirkskrankenhaus Lienz

Vorarlberg - Bitte geben Sie Ihre Institution an

- ◯ Landeskrankenhaus Feldkirch
- ◯ Landeskrankenhaus Hohenems
- ◯ Krankenhaus Dornbirn
- ◯ Landeskrankenhaus Bregenz
- ◯ Landeskrankenhaus Bludenz

Wien - Bitte geben Sie Ihre Institution an

- ◯ AKH Wien
- ◯ Klinik Donaustadt
- ◯ Traumazentrum Wien - Standort Lorenz-Böhler
- ◯ Klinik Ottakring
- ◯ Hanusch-Krankenhaus
- ◯ Traumazentrum Wien - Standort Meidling
- ◯ Klinik Floridsdorf

Gibt es einen „hauseigenen Standard“ in der Versorgung von PatientInnen mit einem Schädel-Hirn-Trauma und der Einnahme einer oralen Antikoagulation / Thrombozytenaggregationshemmern?

- ◯ Ja
- ◯ Nein
- ◯ Sonstige ____________

Create your own automated PDFs with Jotform PDF Editor- It's free

Ist dieser "hauseigene Standard" für alle Abteilungsmitglieder schriftlich vorliegend?

- ◯ Ja
- ◯ Nein

Welche PatientInnen erhalten zur Diagnostik eine Craniale Computertomographie (CCT)?

- ☐ Einnahme von Thrombozytenaggregationshemmern (Acetylsalicylsäure (Thrombo-ASS®), Clopidogrel (Plavix®), Prasugrel (Efient®))
- ☐ Einnahme von Vitamin-K-Antagonisten (Phenprocoumon (Marcoumar®), Acenocoumarol (Sintrom®))
- ☐ Einnahme von Direkten oralen Antikoagulantien (DOAK) (Apixaban (Eliquis®), Edoxaban (Lixiana®), Rivaroxaban (Xarelto®), Dabigatran (Pradaxa®))
- ☐ Anamnestisch regelmäßiger Alkoholabusus / bekannte Alkoholabhängigkeit
- ☐ Bekannte Hämophilie
- ☐ Keine der oben genannten

Bei welchen PatientInnen erfolgt stattdessen eine stationäre Observanz (regelmäßige Vigilanz-, Pupillen-, Blutdruckkontrollen) OHNE Durchführung einer CCT (NICHT wohnhaft in einer Pflegeeinrichtung)?

- ☐ Einnahme von Thrombozytenaggregationshemmern (Acetylsalicylsäure (Thrombo-ASS®), Clopidogrel (Plavix®), Prasugrel (Efient®))
- ☐ Einnahme von Vitamin-K-Antagonisten (Phenprocoumon (Marcoumar®), Acenocoumarol (Sintrom®))
- ☐ Einnahme von Direkten oralen Antikoagulantien (DOAK) (Apixaban (Eliquis®), Edoxaban (Lixiana®), Rivaroxaban (Xarelto®), Dabigatran (Pradaxa®))
- ☐ Anamnestisch regelmäßiger Alkoholabusus / bekannte Alkoholabhängigkeit
- ☐ Bekannte Hämophilie
- ☐ Keine der oben genannten

Bei welchen PatientInnen erfolgt stattdessen eine stationäre Observanz (regelmäßige Vigilanz-, Pupillen-, Blutdruckkontrollen) OHNE Durchführung einer CCT (wohnhaft IN einer Pflegeeinrichtung)?

- ☐ Einnahme von Thrombozytenaggregationshemmern (Acetylsalicylsäure (Thrombo-ASS®), Clopidogrel (Plavix®), Prasugrel (Efient®))
- ☐ Einnahme von Vitamin-K-Antagonisten (Phenprocoumon (Marcoumar®), Acenocoumarol (Sintrom®))
- ☐ Einnahme von Direkten oralen Antikoagulantien (DOAK) (Apixaban (Eliquis®), Edoxaban (Lixiana®), Rivaroxaban (Xarelto®), Dabigatran (Pradaxa®))
- ☐ Anamnestisch regelmäßiger Alkoholabusus / bekannte Alkoholabhängigkeit
- ☐ Bekannte Hämophilie
- ☐ Keine der oben genannten

Zu welchem Zeitpunkt wird die CCT durchgeführt (unter der Annahme, dass diese sofort verfügbar wäre)?

- ◯ Bei Erstbegutachtung
- ◯ Sonstige ________

Bei welchen PatientInnen (NICHT wohnhaft in einer Pflegeeinrichtung) erfolgt nach unauffälliger CCT eine stationäre Observanz (regelmäßige Vigilanz-, Pupillen-, Blutdruck-Kontrollen)?

Create your own automated PDFs with Jotform PDF Editor- It's free

Jotform

- [ ] Einnahme von Thrombozytenaggregationshemmern (Acetylsalicylsäure (Thrombo-ASS®), Clopidogrel (Plavix®), Prasugrel (Efient®))
- [ ] Einnahme von Vitamin-K-Antagonisten (Phenprocoumon (Marcoumar®), Acenocoumarol (Sintrom®))
- [ ] Einnahme von Direkten oralen Antikoagulantien (DOAK) (Apixaban (Eliquis®), Edoxaban (Lixiana®), Rivaroxaban (Xarelto®), Dabigatran (Pradaxa®))
- [ ] Anamnestisch regelmäßiger Alkoholabusus / bekannte Alkoholabhängigkeit
- [ ] Bekannte Hämophilie
- [ ] Keine der oben genannten

Bei welchen PatientInnen (wohnhaft IN einer Pflegeeinrichtung) erfolgt nach unauffälliger CCT eine stationäre Observanz (regelmäßige Vigilanz-, Pupillen-, Blutdruck-Kontrollen)?

- [ ] Einnahme von Thrombozytenaggregationshemmern (Acetylsalicylsäure (Thrombo-ASS®), Clopidogrel (Plavix®), Prasugrel (Efient®))
- [ ] Einnahme von Vitamin-K-Antagonisten (Phenprocoumon (Marcoumar®), Acenocoumarol (Sintrom®))
- [ ] Einnahme von Direkten oralen Antikoagulantien (DOAK) (Apixaban (Eliquis®), Edoxaban (Lixiana®), Rivaroxaban (Xarelto®), Dabigatran (Pradaxa®))
- [ ] Anamnestisch regelmäßiger Alkoholabusus / bekannte Alkoholabhängigkeit
- [ ] Bekannte Hämophilie
- [ ] Keine der oben genannten

Erhalten jene PatientInnen mit unauffälliger CCT, welche zur stationären Observanz aufgenommen wurden, nach 24 Stunden eine routinemäßige Kontroll-CCT?

○ Ja
○ Nein
○ Sonstige ________

Erfolgt bei isoliertem leichten Schädel-Hirn-Trauma routinemäßig die Bestimmung von S100B im Blut?

○ Ja
○ Nein
○ Sonstige ________

Erfolgt bei PatientInnen mit anamnestisch bekanntem regelmäßigen Alkoholabusus/ aus der Krankengeschichte ersichtlicher Alkoholkrankheit eine Blutabnahme und Bestimmung der Gerinnung?

○ Ja
○ Nein
○ Sonstige ________

Create your own automated PDFs with Jotform PDF Editor- It's free
Jotform

Sind Ihnen die "Scandinavian Guidelines for initial management of minimal, mild and moderate head injuries in adults" bekannt?

- ◯ Ja
- ◯ Nein

Arbeiten Sie nach den Guidelines?

- ◯ Ja
- ◯ Nein
- ◯ Sonstige ________

Möchten Sie uns noch etwas mitteilen?

Create your own automated PDFs with Jotform PDF Editor- It's free
Jotform
